# Impact of Prenatal Risk Factors on Congenital Heart Disease in the Current Era

**DOI:** 10.1161/JAHA.113.000064

**Published:** 2013-06-21

**Authors:** Alan Fung, Cedric Manlhiot, Sapna Naik, Herschel Rosenberg, John Smythe, Jane Lougheed, Tapas Mondal, David Chitayat, Brian W. McCrindle, Seema Mital

**Affiliations:** 1 Division of Cardiology, Department of Pediatrics, Hospital for Sick Children, University of Toronto, Toronto, Ontario, Canada (A.F., C.M., S.N., B.W.M.C., S.M.); 2 Department of Pediatrics, London Health Sciences Centre, London, Ontario, Canada (H.R.); 3 Department of Pediatrics, Kingston General Hospital, Kingston, Ontario, Canada (J.S.); 4 Department of Pediatrics, Children's Hospital of Eastern Ontario, Ottawa, Ontario, Canada (J.L.); 5 Department of Pediatrics, Hamilton Health Sciences Centre, Hamilton, Ontario, Canada (T.M.); 6 Department of Obstetrics and Gynecology, The Prenatal Diagnosis and Medical Genetics Program, Mount Sinai Hospital, University of Toronto, Toronto, Ontario, Canada (D.C.)

**Keywords:** congenital heart disease, environment, fetal cardiovascular abnormality, genetic testing, prenatal diagnosis

## Abstract

**Background:**

The healthcare burden related to congenital heart disease (CHD) is increasing with improving survival. We assessed changing trends in prenatal risk factors for CHD in the current era in a Canadian cohort.

**Methods and Results:**

CHD patients <18 years old (n=2339) and controls without structural heart disease (n=199) were prospectively enrolled in an Ontario province‐wide biobank registry from 2008–2011. Family history, frequency of extra‐cardiac anomalies (ECAs), and antenatal risk factors were assessed. Temporal trends were analyzed and associations with CHD were measured using linear and logistic regression. Family history of CHD and frequency of major ECAs was higher in cases versus controls (*P*<0.001). Despite an increase in genetic testing in the recent era, only 9.5% of cases with CHD had a confirmed genetic diagnosis. Yield of genetic testing (ie, frequency of abnormal results) was higher in familial and syndromic cases. There was an increase in parental age at conception, maternal prepregnancy body mass index, maternal urinary tract infections, type 1 diabetes, and exposure to nonfertility medications during pregnancy from 1990–2011. Later year of birth, family history of CHD, presence of major ECAs, maternal smoking during pregnancy, and maternal medication exposure were associated with increased odds of CHD (*P*<0.05 for all). Advanced parental age was associated with increased odds of CHD caused by genetic abnormalities.

**Conclusions:**

The increase in prenatal risk factors for CHD highlights the need for more rigorous ascertainment of genetic and environmental factors including gene‐environment interactions that contribute to CHD.

## Introduction

Congenital heart disease (CHD) is the leading cause of infant deaths due to birth defects.^1^ Despite a strong heritable basis, a genetic etiology is identified in less than 20% of CHD cases.^2^ This reflects a combination of the low sensitivity of routine cytogenetic testing at detecting rare or de novo mutations associated with isolated CHD, and the unmeasured contribution of complex gene‐environment interactions to CHD.^3–4^ Importantly, while the prevalence of acquired heart disease has decreased over the past decade, CHD incidence has remained unchanged and CHD prevalence has increased likely due to better detection and improved survival.^5–8^ In Canada, birth prevalence of CHDs was 10.4 per 1000 total births or 3518 cases as reported by the Canadian Congenital Anomalies Surveillance System in 2007. Extrapolation of these findings estimates currently over 200 000 CHD cases in Canada and over 70 000 in Ontario alone.^9^ The changing population demographics highlight the need to reassess the contribution of genetic and environmental risk factors in the current era.^10^ An exploration of the contribution of noninherited risk factors that are potentially modifiable is particularly important in the context of the growing health burden of CHD.^11^

Reports linking environmental exposures to birth defects have steadily increased during the past decade with reported association of maternal (and paternal) illnesses, nutritional deficiencies, drugs, and chemical exposures during embryonic and early fetal development with CHD. However, a failure to routinely ascertain environmental exposures during pregnancy, difficulty in quantifying these exposures, and maternal recall bias limit the ability to determine causality.^12–17^ The Baltimore‐Washington Infant Study previously associated maternal diabetes, influenza, ibuprofen therapy, parental smoking, and nutritional deficiencies with specific CHD subtypes.^18^ More recent studies have identified other associations including the National Birth Defects Prevention Study that evaluated the potential risk of medication use during pregnancy and birth defects.^16,19–23^ Changes in population demographics (ie, racial or ethnic) and cultural diversity with time may also influence both genetic and environmental risk. We therefore performed a comprehensive analysis of genetic and environmental risk factors in CHD patients enrolled in an Ontario province‐wide biorepository and registry to assess trends in risk factors and their contribution to CHD subtypes in the current era.

## Methods

We established an Ontario province‐wide hospital‐based biobank registry of patients with congenital and other forms of childhood onset heart disease representing the first large‐scale study of CHD in an ethnically diverse population of Southern Ontario. Patients were prospectively enrolled from 6 pediatric and 4 adult cardiac programs across 7 hospitals in the province of Ontario between February 2008 and July 2011 with the goal of studying the genetic and environmental basis of CHD. Inclusion criteria were pediatric patients under the age of 18 years with structural heart disease. Controls without CHD were enrolled concurrently and included patients presenting to the hospital with innocent cardiac murmurs or chest pain with normal echocardiograms, dental lesions, infections, or obesity. The study was approved by the Research Ethics Board of each participating site. Written informed consent was obtained from the patient and/or parent or legal guardian. Consent was obtained for donation of biological samples including DNA, tissue, ongoing review of the patient's medical and surgical records, and completion of an intake questionnaire by the patient/parent. Details of the cohort design and recruitment processes have been previously published.^24^

The following clinical information was captured through questionnaires—maternal and paternal age at conception, parental consanguinity, maternal medical, gestational and obstetric history, smoking, alcohol and drug exposure during first trimester of pregnancy, and a multi‐generational family history of CHD and other birth defects. Exposure was defined by positive maternal history during the first trimester of pregnancy to the various exposures listed in Table 3. First‐, second‐, and third‐degree relatives were defined as previously reported by Oyen et al.^25^ The following information was captured from a review of the patients' medical records: detailed echocardiographic diagnosis (verified by a single observer, SM), results of genetics evaluation, chromosomal, genetic and cytogenetic testing, the presence and type of extra‐cardiac anomalies (ECA) and concurrent medical illnesses. Cases were classified using the International Nomenclature for Congenital Heart Surgery into the following categories: septal defects (SD), endocardial cushion defects (ECD), right heart lesions (RHL), left heart lesions (LHL), transposition of the great arteries (TGA), thoracic vessel anomalies (TVA), patent ductus arteriosus (PDA), laterality disorders (LATDIS), and single ventricles (SV).^26^ ECAs were coded using the World Health Organization's International Classification of Diseases and Related Health Problems 10th Revision (ICD‐10) into major or minor congenital anomaly, non‐congenital anomaly, and chromosomal abnormality. Changes in the frequency from 1990−2011 of genetic screening and of prenatal exposure to noninherited or environmental risk factors were ascertained.

### Statistical Methods

Data are presented as means with standard deviation and frequencies as appropriate. Associations between year of birth and risk factors were evaluated in linear regression models. The differences between annualized frequencies were reported as estimated effect size (EST) with 95% confidence intervals (CI). Factors associated with all CHD and with different lesion subtypes were assessed in logistic regression models using patients without CHD as controls. Associations between exposure and risk were expressed as unadjusted odds ratios (OR) using univariable analysis unless otherwise specified. CHD and its subtypes were compared with controls in univariable and multivariable models using a stepwise algorithm for multivariable model selection (*P*<0.05 to enter). All statistical analyses were performed using SAS statistical software v9.3 (The SAS Institute).

## Results

### Patient Characteristics

**Figure 1.**
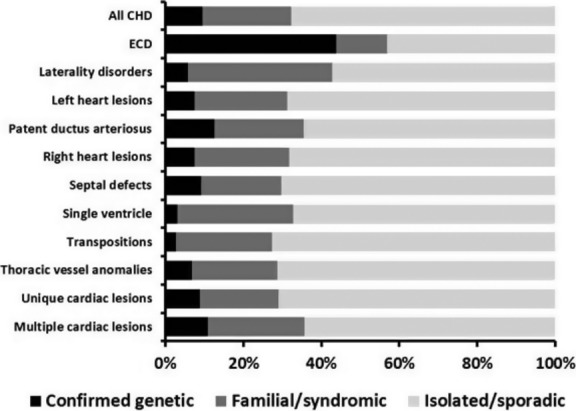
The graph shows proportion of patients with CHD with a confirmed genetic diagnosis or syndrome (black), patients with a positive family history and/or syndromic phenotype without a confirmed genetic diagnosis (dark grey), and patients with isolated, sporadic CHD without a confirmed genetic diagnosis (light grey). The various CHD subtypes are shown on the Y axis. Overall, 9.5% of CHD had a confirmed genetic diagnosis, 21.8% had familial or syndromic CHD without a genetic diagnosis, and 68.7% had isolated or sporadic CHD without a genetic diagnosis. ECDs had the highest frequency of confirmed genetic diagnosis. CHD indicates congenital heart disease; ECD, endocardial cushion defect.

Of 4193 patients with heart disease approached, 3762 subjects consented for enrollment in the biobank (consent rate 90%); 2339 index cases with CHD <18 years old were eligible for this study. An additional 199 children without CHD were recruited as controls (consent rate 89%). A total of 1581 (68%) patients had a single primary lesion, 758 (32%) had diagnoses encompassing multiple cardiac lesions. This is consistent with findings in the National Birth Defect Prevention Study.^27^ Distribution of primary and secondary CHD diagnoses are listed in Tables 1 and 2, respectively. Racial distribution for CHD cases was 79% white, 15% Asian, 5% black, and 1% other, and for controls was 71% white, 22% Asian, and 7% black (*P*<0.05 versus cases). Racial distribution differed between cases and controls but was comparable to Ontario population census data from 2006 (76% white, 20% Asian, 3% black, 1% other).^28^ Gender distribution was similar between controls and cases (60% versus 57% males respectively, *P*=0.41). However, there was a preponderance of males to females in the following CHD subtypes relative to controls: ECD (OR 1.6 [1.1 to 2.4], *P*=0.03), PDA (OR 1.6 [1.1 to 2.3], *P*=0.01), SD (OR 1.5 [1.1 to 2.1], *P*=0.01) and TVA (OR 1.5 [1.0 to 2.2], *P*=0.05). Of the 2339 CHD index cases, 9.5% had a confirmed genetic diagnosis or recognizable syndrome, 21.8% had a positive family history of CHD or associated major ECAs of unknown etiology, and the remaining 68.7% had isolated, sporadic CHD of unknown etiology (ie, negative family history of CHD and/or no associated ECAs) (Figure 1). The frequency of confirmed genetic diagnosis was highest for ECDs (44% of ECDs had a confirmed genetic diagnosis) and lowest for TGA and SV (only 3% had a confirmed genetic diagnosis).

**Table 1.** Congenital Heart Disease Subtypes by Primary Diagnostic Categories

	N (%)	Single Diagnosis, %	Multiple Diagnoses, %
Endocardial cushion defects	176 (7.5)	50.6	49.4
Laterality disorders	35 (1.5)	34.3	65.7
Left heart lesions	766 (32.7)	65.3	34.7
Patent ductus arteriosus	288 (12.3)	9.7	90.3
Right heart lesions	784 (33.5)	53.2	46.8
Septal defects	730 (31.2)	44.9	55.1
Single ventricle	64 (2.7)	14.1	85.9
Transposition of great arteries	392 (16.8)	34.4	65.6
Thoracic vessel anomalies	206 (8.8)	30.6	69.4

**Table 2.** CHD Subtypes by Primary and Secondary Diagnostic Categories (n=2339 CHD cases)

Diagnosis	N	%
Isolated LHL	500	21.4
Isolated RHL	417	17.8
Isolated SD	328	14.0
Isolated TGA	135	5.8
Isolated ECD	89	3.8
Isolated TVA	63	2.7
Isolated PDA	28	1.2
Isolated LATDIS	12	0.5
Isolated SV	9	0.4
SD+RHL	89	3.8
SD+LHL	56	2.4
SD+TGA+PDA	44	1.9
RHL+TVA	38	1.6
RHL+PDA	37	1.6
LHL+PDA	27	1.2
RHL+TGA	25	1.1
SD+LHL+PDA	25	1.1
SD+TGA	24	1.0
SD+RHL+TGA	24	1.0
ECD+LHL	22	0.9
SD+RHL+PDA	22	0.9
LHL+TGA	19	0.8
SD+TVA	18	0.8
SD+PDA	16	0.7
SD+RHL+LHL	15	0.6
ECD+PDA	14	0.6
RHL+LHL	14	0.6
TGA+PDA	12	0.5
RHL+LHL+TGA	12	0.5
ECD+RHL	11	0.5
RHL+TGA+SV	6	0.3
SD+LHL+TGA	6	0.3
RHL+TVA+PDA	6	0.3
LHL+TGA+SV	5	0.2
SD+TVA+PDA	5	0.2
RHL+TGA+PDA	5	0.2
RHL+TGA+TVA	5	0.2
SD+LHL+TGA+PDA	5	0.2
SD+RHL+TGA+PDA	5	0.2
Other combinations	146	6.2

CHD indicates congenital heart disease; LHL, left heart lesions; RHL, right heart lesions; SD, septal defects; TGA, transposition of great arteries; ECD, endocardial cushion defect; TVA, thoracic vessel anomalies; PDA, patent ductus arteriosus; LATDIS, laterality disorder; SV, single ventricles.

### Familial CHD

**Figure 2.**
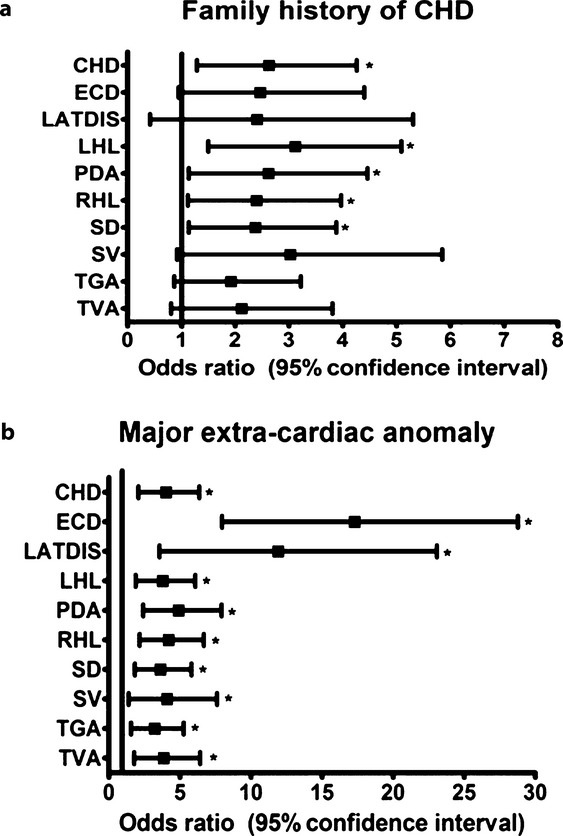
a, Patients with CHD had higher odds of having affected family members with CHD. Associations were significant for overall CHD and specifically for subtypes of LHL, RHL, PDA and SD. b, Patients with CHD had higher odds of having major extra‐cardiac anomalies. Associations were significant for all CHD subtypes except LATDIS (*P<0.05 vs controls). The X axis shows the odds ratio with 95% confidence intervals for association. The Y axis shows the various CHD subtypes. CHD indicates congenital heart disease; ECD, endocardial cushion defect; LATDIS, laterality disorder; LHL, left heart lesions; PDA, patent ductus arteriosus; RHL, right heart lesions; SD, septal defects; SV, single ventricles; TGA, transposition of great arteries; TVA, thoracic vessel anomalies.

CHD recurrence was assessed through detailed family pedigrees in all subjects. Parental consanguinity was reported in 3.5% of cases with CHD which was comparable to controls. A 3‐generation family history revealed a 2‐fold higher frequency of any affected family member with CHD in cases compared to controls (18% versus 9% respectively, *P*=0.005) and higher frequency of affected first‐degree relatives in cases compared to controls (9% versus 5%, respectively) (Table 3). The lesions most strongly associated with a positive family history were LHL (OR 2.8 [1.5 to 5.1], *P*=0.001), PDA (OR 2.3 [1.1 to 4.5], *P*=0.02), RHL (OR 2.1 [1.1 to 3.9], *P*=0.02), and SD (OR 2.1 [1.1 to 3.9], *P*=0.02) (Figure 2a). Twenty‐three percent of cases with a positive family history underwent genetic testing; overall positive yield was 32% (ie, 32% of all cases tested showed abnormalities).

**Table 3.** Family History and Extracardiac Anomalies in CHD cases and Controls

	Controls (n=199)	CHD (n=2339)	Odds Ratio*	*P* Value
Parental consanguinity	6/154 (3.9%)	64/1575 (4.1%)	1.0 (0.4 to 2.7)	1.00
Family history of CHD	14/153 (9.2%)	292/1633 (17.9%)	2.2 (1.2 to 4.0)	0.005
First‐degree relatives only	7/152 (4.6%)	133/1533 (8.7%)	2.0 (0.9 to 4.7)	0.14
Minor or major congenital anomalies	16/177 (9.0%)	589/2159 (27.3%)	3.8 (2.2 to 6.6)	<0.001
Minor congenital anomalies	8/177 (4.5%)	267/2159 (12.4%)	2.9 (1.4 to 6.6)	<0.001
Major congenital anomalies	12/177 (6.8%)	484/2159 (22.4%)	4.0 (2.1 to 7.6)	<0.001
Nervous system	2/177 (1.1%)	29/2159 (1.3%)	1.9 (0.3 to 7.3)	1.00
Eye, ear, face and neck	1/177 (0.6%)	96/2159 (4.5%)		0.009
Respiratory system	2/177 (1.1%)	40/2159 (1.9%)	1.7 (0.4 to 10.0)	0.77
Cleft lip and cleft palate	0/177 (0.0%)	21/2159 (1.0%)		0.40
Digestive system	0/177 (0.0%)	58/2159 (2.7%)		0.02
Genital organs	1/177 (0.6%)	21/2159 (1.0%)		1.00
Urinary system	4/177 (2.3%)	21/2159 (1.0%)	0.4 (0.1 to 1.5)	0.12
Musculoskeletal system	7/177 (4.0%)	112/2159 (5.2%)	1.3 (0.6 to 3.2)	0.59
Other malformations	4/177 (2.3%)	74/2159 (3.4%)	1.5 (0.5 to 5.0)	0.52
Chromosomal abnormalities	0/199 (0.0%)	223/2339 (9.5%)		<0.001
Abnormal genetic results	1/167 (0.6%)	97/2005 (4.8%)		0.006

CHD indicates congenital heart disease.

* Odds ratios were only calculated for 2 or more events.

### Syndromic CHD

Syndromic phenotype was defined as having one or more major ECAs. There was a 3‐fold higher frequency in cases versus controls for both major and minor ECAs (27% versus 9% respectively, OR 3.6 [2.1 to 6.4], *P*<0.001) (Table 3) and strong association of ECAs with all CHD subtypes. The frequency of ECAs, as expected, was highest with ECDs (51%, *P*<0.001) and LATDIS (34%, *P*=0.007) compared with other CHD subtypes (18%) (Figure 2b). Major ECA categories are outlined in Table 3. Facial, oral, and digestive system abnormalities were the most commonly associated ECAs with various CHD subtypes. Thirty‐nine percent of cases with a syndromic phenotype underwent genetic testing of which 43% cases had abnormal test results (Table 4). Forty‐one percent of those tested had trisomy 21 (4.1% of total cohort) and 19% of those tested had 22q11.2 deletion syndrome (1.7% of total cohort).

**Table 4.** Syndromes Associated With CHD

Syndrome	Frequency	%
Alagille syndrome	3	0.12
Arnold‐Chiari Syndrome	1	0.04
Blackfan‐Diamond Syndrome	1	0.04
CHARGE	4	0.16
Chromosome 9p deletion	1	0.04
DiGeorge (22q deletion) Syndrome	43	1.69
Ehlers Danlos Syndrome	2	0.08
Goldenhar Syndrome	4	0.16
Holt‐Oram Syndrome	1	0.04
Jacobsen Syndrome	1	0.04
Kartagener Syndrome	1	0.04
Klinefelter's Syndrome	1	0.04
Klippel‐Feil	1	0.04
LEOPARD Syndrome	1	0.04
Loeys‐Dietz syndrome	2	0.08
Marfan syndrome	2	0.08
Neurofibromatosis	2	0.08
Noonan Syndrome	15	0.59
OHDO Blepharophimosis Syndrome	1	0.04
Other	5	0.20
Rett Syndrome	1	0.04
Scheie's syndrome	1	0.04
Trisomy 13	1	0.04
Trisomy 21 (Down syndrome)	105	4.14
Turner syndrome	9	0.35
VACTERL	3	0.12
VATER	1	0.04
WAGR Syndrome	1	0.04
Wiedemann‐Rauterstrauss	1	0.04
Williams Syndrome	9	0.35
Wiskott‐Aldrich Syndrome	2	0.08
Total	226	8.9

CHD indicates congenital heart disease CHARGE, Coloboma of eyes, Heart defects, Atresia of nasal choanae, Retardation of growth and development, Genitourinary anomalies, Ear abnormalities and deafness; LEOPARD, Lentigenes, ECG conduction abnormalities, Ocular hypertelorism, Pulmonic stenosis, Abnormal genitalia, Retardation of growth, sensorineural Deafness; VACTERL, Vertebral anomalies, imperforate Anus, Cardiac anomalies, Tracheo‐Esophageal fistula, growth Retardation, Limb anomalies; VATER, Verterbral anomalies, imperforate Anus, Tracheo‐Esophageal fistula, Retardation of growth; WAGR, Wilms tumor, Aniridia, Genitourinary anomalies, mental Retardation.

### Increased Frequency of Genetic Testing in the Current Era

**Figure 3.**
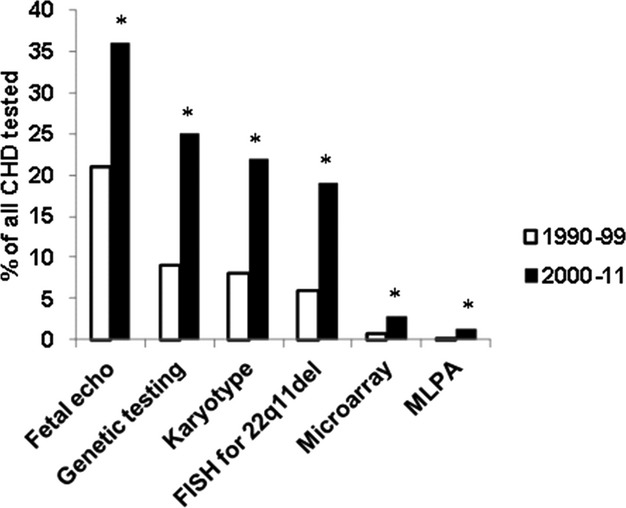
Change in frequency of fetal echocardiographic screening and genetic and cytogenetic testing in CHD cases from 1990–2011. *P<0.001 between 2000–11 vs 1990–1999. CHD indicates congenital heart disease; FISH, fluorescent in situ hybridization; MLPA, multiplex ligation‐dependent probe amplification.

During 1990 to 2011, frequency of fetal echocardiographic screening increased from 21% (1990–99) to 36% (2000–11) (EST: +1.7% [1.3%, +2.2%]/year, *P*<0.001). During the same period, frequency of genetic testing increased from 9% to 25% (EST: +1.4% [+1.1%, +1.6%]/year, *P*<0.001) including increased karyotype testing, fluorescent in situ hybridization (FISH) for 22q11deletion, microarray testing, and multiplex ligation‐dependent probe amplification (MLPA) for detecting deletions/duplications (*P*<0.01 for all) (Figure 3). This was associated with increased diagnoses of cytogenetic abnormalities from 2.7% to 5.3% in the overall cohort (EST: +0.3% [+0.2%, +0.5%]/year, *P*<0.001). However, the increase in frequency of genetic testing was not paralleled by an increase in the yield from genetic tests (EST: +0.2% [−0.7%; +1.0%]/year, *P*=0.71). Thus, the proportion of patients with a clinically diagnosed syndrome and/or abnormal genetic test in the overall cohort only increased from 8% to 11% (EST: +0.6 [+0.4%; +0.8%]/year, *P*<0.001) representing a minor fraction of all CHD despite a 3‐fold increase in testing indicating that the increase in confirmed genetic diagnoses was due to increased testing rather than improved sensitivity of the genetic tests. Overall, the yield on genetic testing was highest in syndromic patients (43%) followed by familial cases (32%) and lowest in isolated, sporadic CHD (8%) using conventional testing methods.

### Increased Prenatal Risk Factor Burden in the Recent Era

**Figure 4.**
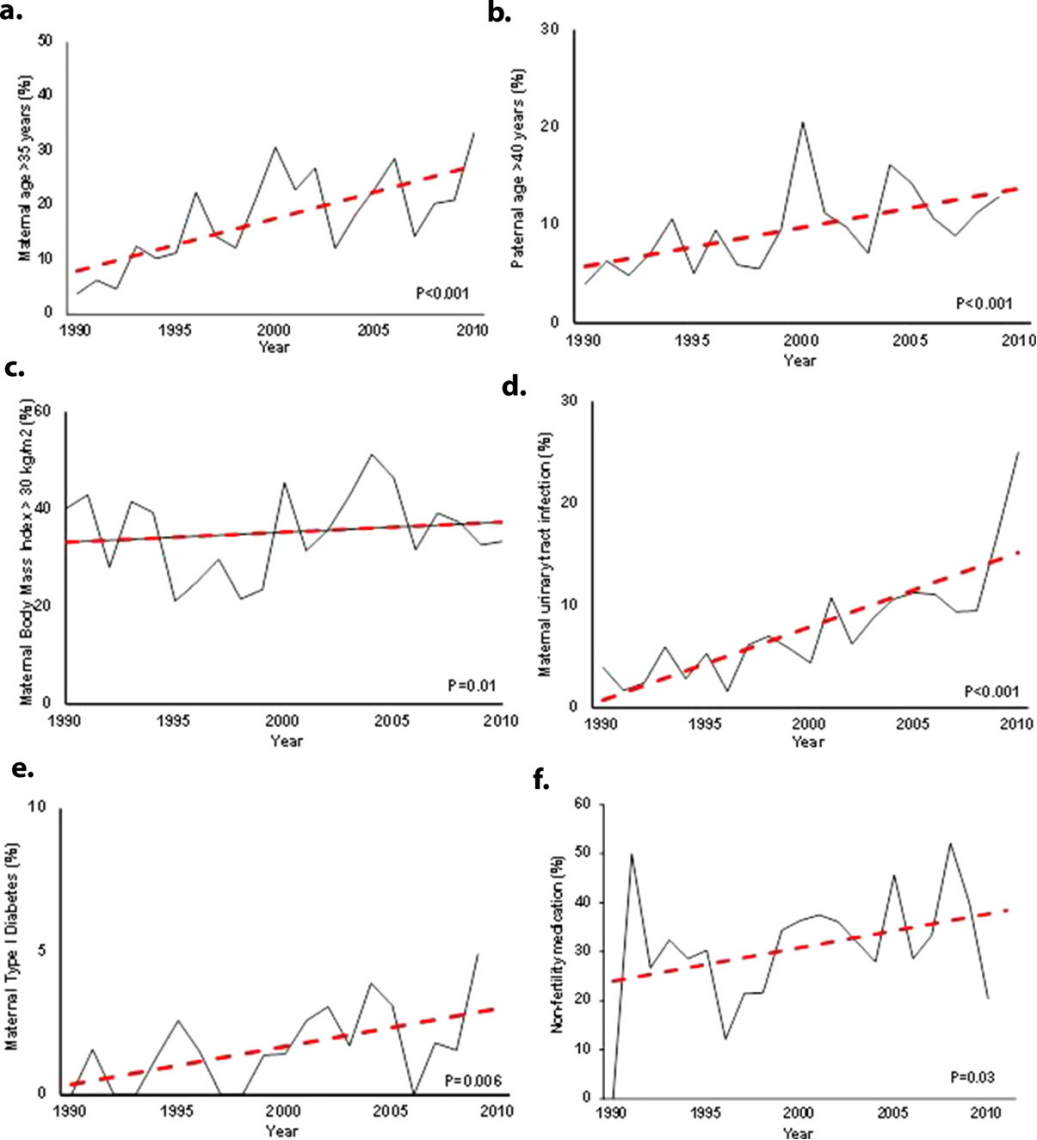
Change in frequency of prenatal risk factors in CHD cases from 1990–2011. a, CHD cases with history of maternal age at conception >35 years increased from 1990–2011 (*P*<0.001). b, CHD cases with history of paternal age at conception >40 years increased from 1990–2011 (*P*<0.001). c, CHD cases with pre‐pregnancy maternal body mass index >30 kg/m^2^ increased from 1990–2011 (*P*=0.01). d, CHD cases with history of maternal urinary tract infections during early pregnancy increased from 1990–2011 (*P*<0.001). e, CHD cases with history of pre‐pregnancy maternal type 1 diabetes increased from 1990–2011 (*P*=0.006). f, CHD cases with history of maternal non‐fertility medication use during early pregnancy increased from 1990–2011 (*P*=0.03). CHD indicates congenital heart disease.

We assessed prenatal risk factors through data from questionnaires (Table 5). Prenatal risk factor exposure was identified in 53% of subjects with overall increase in frequency of prenatal exposures from 1990–99 to 2000–11 assessed using linear regression models. These were factors previously known to be associated with birth defects and additional factors identified through questionnaire reports (Figure 4). There was an increase in maternal age at conception (29±5 to 31±6 years, EST: +6 [4 to 9] weeks/year, *P*<0.001), paternal age at conception (31±6 to 33±7 years, EST: +6 [3 to 9] weeks/year, *P*<0.001), increase in pre‐pregnancy maternal body mass index (BMI) (23.8±5.0 to 24.9±6.0, EST: +0.07 [0.02 to 0.13] kg/m^2^ per year, *P*=0.01), frequency of maternal urinary tract infections during pregnancy (5% to 9%, EST: +0.5% [+0.2%, +0.7%]/year, *P*<0.001), type 1 diabetes (0.8% to 2.8%, EST: +0.2% [+0.1%, +0.3%]/year, *P*=0.006), and exposure to nonfertility medications during the first trimester (23% to 33%, EST: +0.7% [+0.1%, +1.3%]/year, *P*=0.03). Frequency of maternal smoking (23% to 16%, EST: −0.7% [−0.3%, −1.0%]/year, *P*<0.001) and alcohol ingestion during early pregnancy (9% to 7%, EST: −0.2% [−0.0%, −0.5%]/year, *P*=0.06) declined with time. There was no change over time in the frequency of rubella, hypertension, in vitro fertilization, gestational diabetes, and recreational drug use.

**Table 5.** Prenatal Risk Factors in Controls and CHD Patients

	Controls (n=199)	CHD (n=2339)	Odds Ratio*	*P* Value
Paternal age at conception, y	32.2±6.4	32.5±6.4	0.99 (0.97 to 1.02)	0.60
Maternal factors				
Maternal age at conception, y	29.9±5.5	29.4±5.2	1.02 (0.99 to 1.05)	0.26
Prepregnancy BMI, kg/m^2^	24.4±5.5	24.6±5.8	0.99 (0.95 to 1.05)	0.66
Type 1 diabetes mellitus	3/157 (1.9%)	27/1547 (1.8%)	0.9 (0.3 to 3.8)	0.75
In vitro fertilization	2/144 (1.4%)	20/1061 (1.9%)	1.4 (0.3 to 8.5)	1.00
Maternal environmental exposures				
Smoking	18/155 (11.6%)	319/1508 (21.2%)	2.0 (1.2 to 3.5)	0.004
Alcohol consumption	12/155 (7.7%)	125/1496 (8.4%)	1.1 (0.6 to 2.1)	0.88
Recreational drug use	5/153 (3.3%)	60/1484 (4.0%)	1.2 (0.8 to 3.6)	0.83
General anesthesia	1/151 (0.7%)	32/1471 (2.2%)		0.36
Fertility medications	0/140 (0.0%)	35/611 (5.7%)		0.001
Nonfertility medications	24/140 (17.1%)	190/611 (31.1%)	2.2 (1.3 to 3.6)	<0.001
Analgesics	2/140 (1.4%)	25/611 (4.2%)	2.9 (0.7 to 18.2)	0.20
Antihypertensives	0/140 (0.0%)	8/611 (1.3%)		0.38
Antibiotics	1/140 (0.7%)	9/611 (1.5%)		0.70
Antiemetics	2/140 (1.4%)	27/611 (4.5%)	3.2 (0.7 to 19.6)	0.14
Antihistamines	0/140 (0.0%)	3/611 (0.5%)		0.16
Antiinflammatory/NSAIDs	3/140 (2.1%)	8/611 (1.3%)	0.6 (0.1 to 2.9)	0.44
Bronchodilators	1/140 (0.7%)	9/611 (1.5%)		0.70
Hormones	5/140 (3.6%)	46/611 (7.5%)	2.2 (0.8 to 6.4)	0.13
Psychoactive agents	3/140 (2.1%)	17/611 (2.8%)	1.3 (0.4 to 5.6)	1.00
Vaccinations	1/140 (0.7%)	2/611 (0.3%)		0.46
Known teratogenic medications	4/140 (2.9%)	21/611 (3.4%)	1.2 (0.4 to 4.2)	1.00
Pregnancy complications				
Polyhydramnios	2/139 (1.4%)	29/1278 (2.3%)	1.6 (0.4 to 9.7)	0.76
Oligohydramnios	3/140 (2.1%)	38/1273 (3.0%)	1.4 (0.4 to 5.8)	0.79
Gestational diabetes	8/155 (5.2%)	81/1536 (5.3%)	1.0 (0.5 to 2.3)	1.00
Hypertension	16/149 (10.7%)	135/1514 (8.9%)	0.8 (0.6 to 1.5)	0.45
Infection	14/155 (9.0%)	190/1559 (12.2%)	1.5 (0.8 to 2.8)	0.30
Fever	5/154 (3.3%)	46/1462 (3.1%)	1.0 (0.4 to 2.8)	0.81
Rubella	0/151 (0.0%)	6/1523 (0.4%)		0.48
Urinary tract infection	8/152 (5.3%)	106/1500 (7.1%)	1.4 (0.6 to 3.1)	0.50
Other viral illness	9/150 (6.0%)	52/1451 (3.6%)	0.6 (0.3 to 1.3)	0.17

CHD indicates congenital heart disease; BMI, Body mass index; NSAID, nonsteroidal antiinflammatory drug.

* Odds ratios were only calculated for 2 or more events.

### Association of Environmental Risk Factors With CHD

**Figure 5.**
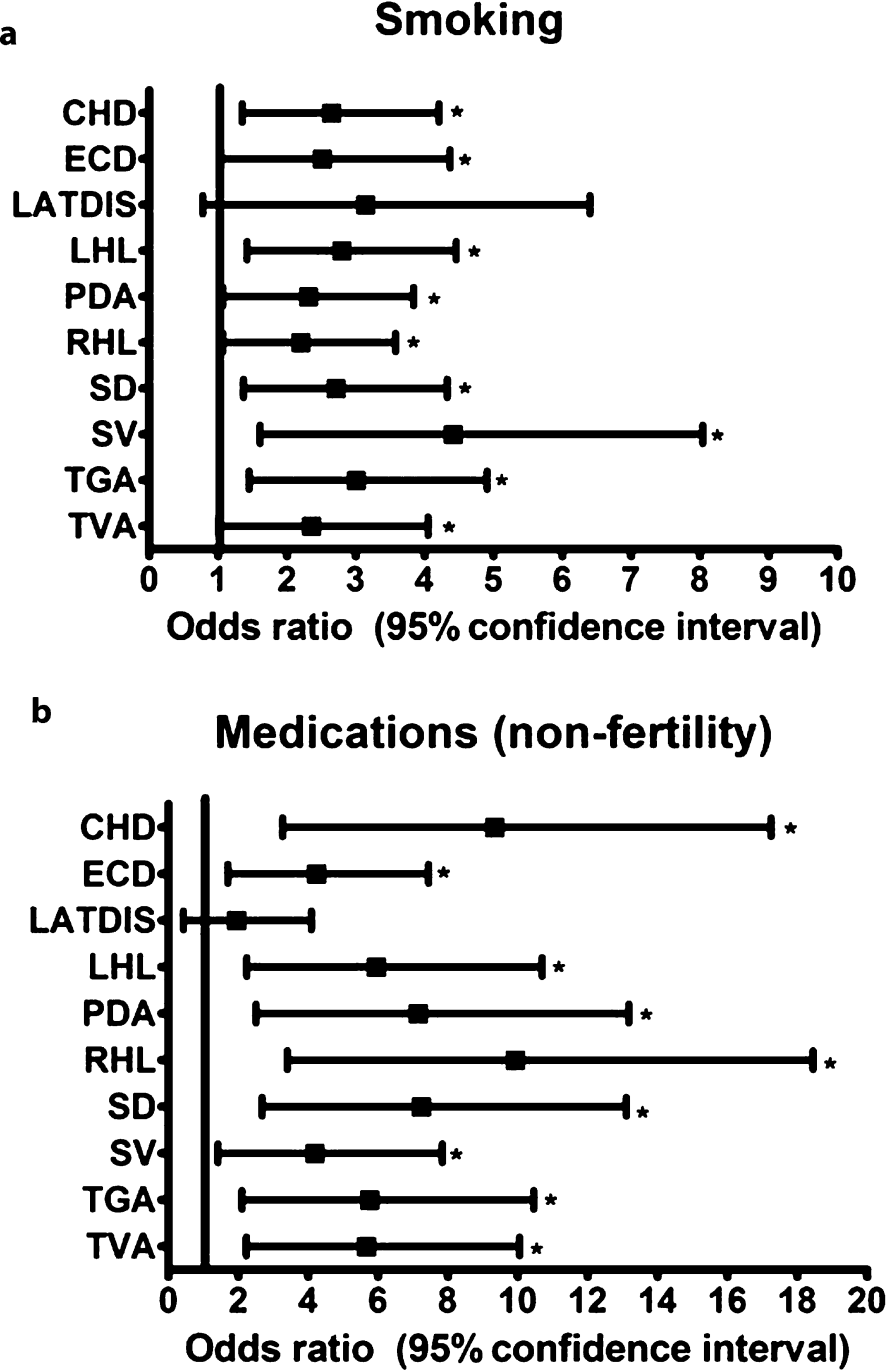
a, Patients with CHD had higher odds of having a maternal history of smoking during pregnancy. Associations using logistic regression were significant for all CHD except LATDIS. *P<0.05 vs controls. b, Patients with CHD had higher odds of having a maternal history of intake of non‐fertility medications during pregnancy. Associations were significant for all CHD subtypes except LD. *P<0.05 vs controls. The X axis shows the odds ratio with 95% confidence intervals for association. The Y axis shows the various CHD subtypes. CHD indicates congenital heart disease; ECD, endocardial cushion defect; LATDIS, laterality disorder; LHL, left heart lesions; PDA, patent ductus arteriosus; RHL, right heart lesions; SD, septal defects; SV, single ventricles; TGA, transposition of great arteries; TVA, thoracic vessel anomalies.

We compared the frequency of prenatal risk factors including medication exposures between CHD cases and controls (Table 5). Medications during pregnancy were classified by safety profile: 55% had a known safety profile, 11% were known teratogens, and 34% had an unknown safety profile (including 5.7% fertility drugs). The factors most strongly associated with risk of CHD were maternal smoking and exposure to fertility and nonfertility medications during the first trimester. All CHD subtypes except LATDIS showed a positive association with maternal smoking and with maternal exposure to nonfertility medications (Figure 5a, 5b). Advanced parental age was seen in association with CHD with genetic abnormalities. We were unable to generate ORs for fertility medications since there were no exposures in controls.

### Multivariable Analysis

**Figure 6.**
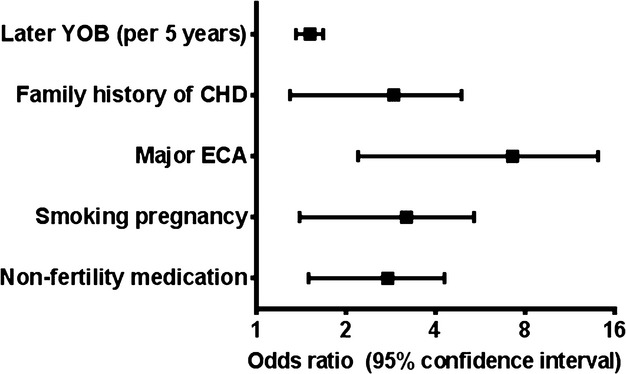
Risk factors associated with CHD on multivariable analysis were later year of birth, positive family history of CHD, presence of major ECA, maternal smoking during pregnancy, and maternal intake of non‐fertility medications during pregnancy. YOB indicates year of birth; CHD, congenital heart disease; ECA, extra‐cardiac anomalies.

In multivariable regression models (χ^2^=70.2, *P*<0.0001; c‐statistic=0.74), the following factors were associated with CHD: later year of birth (OR: 1.09 [1.04 to 1.13]/year, *P*<0.001), family history of CHD (OR: 2.5 [1.3 to 4.9], *P*=0.006), presence of major ECAs (OR: 5.5 [2.2 to 14.1], *P*<0.001), maternal smoking during pregnancy (OR: 2.8 [1.4 to 5.4], *P*=0.003), and maternal exposure to nonfertility medications (OR: 2.5 [1.5 to 4.3], *P*<0.001) (Figure 6). These associations were seen across the cohort independent of family history or syndromic phenotype. The association of older maternal age (OR: 1.18 [1.06 to 1.30] per 10‐year increase in age at conception, *P*=0.002) and older paternal age (OR: 1.10 [1.01 to 1.20] per 10‐year increase in age at conception, *P*=0.02) was limited to CHD patients with genetic abnormalities.

## Discussion

In a study of genetic and environmental risk factors in a prospectively enrolled cohort of children with CHD from a multiethnic population in Ontario, we identified a high proportion of inherited CHD but low sensitivity of current genetic testing methods in identifying genetic loci for CHD particularly in isolated or sporadic CHD. Our findings also demonstrated a rising trend in prenatal environmental exposures in the recent era and a strong association of these factors with CHD.

The first important finding was the high prevalence of CHD in first‐degree relatives (9%) compared to that reported in a recent Danish cohort study by Oyen et al^25^ who reported a 3.1% prevalence of CHD in first‐degree relatives. Previous cohort studies have revealed CHD recurrence rates between 2.7% and 4.1% with higher recurrence rates in some subtypes (eg, 8% in syndromic left ventricular outflow tract obstructions).^29^ While the higher recurrence rate identified in our cohort may reflect differences in heritability associated with different ethnicities, it may also represent better ascertainment of family history which was done prospectively as opposed to through a retrospective review of registry data. Our findings emphasize the importance of routine ascertainment of 3‐generation family history as was emphasized in the American Heart Association guidelines.^3^ However, family history alone may miss “silent” cardiac lesions like bicuspid aortic valve, small SDs, or minor TVAs and thereby underestimate familial incidence. Routine echocardiographic screening of family members particularly for lesions with a high recurrence risk may better help identify familial cases and help prioritize these cases for genetic testing given the higher yield of genetic testing in familial CHD. Importantly, since yield on genetic testing is higher in familial compared to sporadic cases, ascertainment of family history will help prioritize these cases for genetic evaluation.

The relatively high burden of ECAs in our cohort was comparable to findings in Danish and Norwegian cohorts.^25,30^ The utility of classifying CHDs by cardiac and extra‐cardiac phenotypes was shown to be a useful approach for deciphering disease etiology in a report by Botto et al^27^ using data from the National Birth Defect Prevention Study. Relatively high prevalence of ECAs, particularly major ECAs (22%), not only in cases with ECDs and LD as would be expected, but also in other CHD subtypes reinforces the importance of detailed physical examination and screening of CHD cases for ECAs. Knowledge of extra‐cardiac phenotype is useful in prenatal and postnatal counseling due to the potentially adverse impact of major ECAs on overall outcomes. More importantly, early screening and identification of ECAs can facilitate preemptive and perioperative management strategies that reduce the adverse impact of unrecognized anomalies.^31–32^ Also, given the higher yield on genetic testing of syndromic cases, this approach may also help identify a subset of patients who should be offered genetic testing.

Disappointingly, despite a high proportion of inherited and/or syndromic CHD and overall increase in genetic testing in patients with CHD in the current era, genetic diagnoses were confirmed in only 9.5% of patients. Overall yield of genetic testing even in the current era was low at 20%. While the yield can be increased by targeting genetic testing to patients with familial or syndromic CHD that comprise nearly 40% of the CHD cohort, current testing methods remain relatively insensitive in their ability to identify small structural variations and/or rare sequence variations in coding and noncoding regions that likely contribute to a large proportion of CHD. High‐resolution genomic arrays and next‐generation sequencing‐based diagnostics are likely to become important tools in identifying these single or compound rare and de novo variants particularly in patients with sporadic isolated CHD who comprise the vast majority of CHD.^33–35^ These technologies are rapidly becoming available in the clinical arena and are likely to improve the yield of genetic tests as well as improve our ability to identify gene‐gene interactions and a “polygenic” etiology, while permitting better discrimination between benign and pathogenic variants.

The search for the genomic basis of complex disorders is incomplete without an assessment of environmental factors that, either independently or through gene‐environment interactions, can influence disease causation. We found a significant increase in the frequency of prenatal exposure to environmental risk factors in the current era. These included advanced maternal and paternal age at conception, higher maternal BMI, higher frequency of maternal medical conditions during pregnancy like urinary tract infections and type 1 diabetes, and higher prenatal exposure to medications. Although maternal smoking and alcohol consumption rates decreased over time, maternal smoking remained highly associated with all types of CHD. On multivariable analysis, later year of birth, positive family history of CHD, presence of major ECAs, maternal smoking, and maternal intake of medications remained associated with the risk of CHD (Figure 6). Unlike some previously published studies, we did not find a significant association of CHD with maternal obesity or maternal alcohol consumption on multivariable analysis.^11,18,36–37^ This may reflect the decrease in maternal alcohol consumption and lower prevalence of obesity in our cohort compared to other cohorts and/or the cosegregation of these risk factors with other risk factors identified in our study.^15,17^ A particularly important finding was the association of advanced parental age with CHD with genetic abnormalities consistent with previously reported associations of advanced maternal age with aneuploidy and advanced paternal age with germline mutations, and more recently with de novo mutations in disorders like childhood autism.^38^ This finding raises concerns about the mutational burden associated with rising parental age on CHD and emphasizes the need for routine prenatal or postnatal screening with genetic testing and echocardiography in pregnancies with advanced parental age.

A factor that emerged significantly associated with all forms of CHD independent of the presence of genetic abnormalities was maternal intake of fertility and nonfertility medications. This was concerning particularly in light of the increase in medication use during pregnancy in our cohort. Our findings are concordant with a recent study from Australia which reported a higher frequency of birth defects in pregnancies with assisted conception (8.3%) versus 5.8% birth defects in pregnancies not involving assisted conception (OR 1.47).^39^ Our findings are also consistent with other studies which reported a significant association between fertility medication use and CHD in a recent National Birth Defects Prevention Study report that demonstrated an association of septal heart defects and coarctation of aorta with prenatal clomiphene citrate intake.^40–41^ The association with nonfertility medications is equally concerning and highlights the gaps in our knowledge of the safety of commonly used drugs during pregnancy. We recognize that these associations do not necessarily indicate causality and, therefore, caution is advised when making generalized inferences of causation. Also it is not clear whether the association with CHD is related to the use of medication or to the underlying medical condition for which medications were used. Nonetheless, our findings raise concerns about the presumed safety of prescribed or over‐the‐counter medications and highlight the need for more rigorous assessment of the safety of common medications used during pregnancy. They also suggest that consideration should be given to early screening of pregnancies involving assisted conception for CHD. Finally, an improved knowledge of pharmacogenomics may enable us in the future to identify which pregnancies are susceptible to the teratogenic effects of specific medications and help individualize prenatal counseling towards reducing risk factor exposure in at‐risk pregnancies.^42^ The importance of population biorepositories such as ours that capture both genetic and environmental data to address these gaps in knowledge cannot be overemphasized.^24^

Our study was limited by the lack of routine echocardiographic, extra‐cardiac, and genetic screening of all CHD families which may have resulted in underestimation of recurrence rates and of ECAs. Since patients were recruited after birth, the study excluded prenatally diagnosed cases that died or underwent termination antenatally or those that died early postnatally which may have resulted in underrepresentation of the most severe phenotypes. However, as a case cohort study to analyze risk factors for CHD, the study cohort is still considered representative of postnatal CHD survivors. The relatively small size of the control cohort may have reduced our power to detect some significant associations and resulted in some OR being calculated from rare events in controls. Where possible, factors were grouped into categories (eg, nonfertility medications) to increase event rate and improve the precision of OR calculations. Prevalence of consanguinity in cases and controls was relatively high compared to a previous Canadian study which utilized data from 1959 and therefore did not account for the ethnic diversity of Ontario, immigration of ethnic populations, nor increased consanguinity in Asian and South Asian populations.^43^ Another limitation was the lack of ascertainment of socio‐economic and educational status, and the reliance on questionnaires rather than blood sampling of pregnant mothers to assay environmental exposures that can result in recall bias. Nonetheless, coupling detailed assessment of both prenatal and environmental risk factors with sample collection for genomics research has enabled us to identify a cohort of high‐risk women with prenatal exposures that will be explored further to define the gene‐environment interactions that contribute to complex multifactorial disorders like CHD.

## Conclusion

In summary, our study highlights the growing burden of genetic and environmental prenatal risk factors for CHD in the current era, in particular the rising prevalence of maternal obesity and diabetes, advanced maternal and paternal age at conception, and increased use of medications. It highlights a pressing need for better surveillance for CHD both antenatally and postnatally, routine ascertainment and counselling regarding environmental exposures during pregnancy, routine family history in all affected pregnancies, and better genetic screening technology. Public health advocacy measures to reduce environmental risk especially for modifiable risk factors like smoking, alcohol, obesity, diabetes, and medication use are important. Consideration should be given to routine antenatal screening for CHD in women with multiple risk factors including advanced parental age, preexisting maternal conditions and maternal exposure to known teratogens and fertility drugs. Ultimately, this knowledge can be used for early prevention, early risk stratification, and the development of targeted diagnostics and therapies to reduce the health and economic burden of CHD.
